# The pathogenesis of HIV infection: stupid may not be so dumb after all

**DOI:** 10.1186/1742-4690-3-60

**Published:** 2006-09-08

**Authors:** Stephen M Smith

**Affiliations:** 1 Saint Michael's Medical Center and The New Jersey Medical School, Newark New Jersey 07102, USA

## Abstract

In the mid-1990's, researchers hypothesized, based on new viral load data, that HIV-1 causes CD4^+ ^T-cell depletion by direct cytopathic effect. New data from non-human primate studies has raised doubts about this model of HIV-1 pathogenesis. Despite having high levels of viremia, most SIV infections are well tolerated by their natural hosts. Two recent studies of these models provide information, which may be useful in determining how HIV-1 causes CD4^+ ^T-cell loss. A full understanding of pathogenesis may lead to novel therapies, which preserve the immune system without blocking virus replication.

## Discussion

HIV-1 infection is characterized by an insidious deterioration of the cellular immune system[1]. Both the quantity and proportion of plasma CD4^+ ^T-cells decrease steadily over a period of years to decades, and this progressive loss of CD4^+ ^T-cells is associated with the development of acquired immunodeficiency syndrome (AIDS) in infected individuals. The degree of immunodeficiency associated with HIV-1 infection, as defined by the onset of opportunistic diseases, correlates closely with plasma CD4^+ ^T-cell counts. Moreover, the rate at which immunosuppression develops also closely reflects the levels of HIV-1 RNA in plasma, such that the higher the HIV-1 viral load, the greater the loss of circulating CD4^+ ^T-cells per year. A decade ago, researchers believed that the CD4^+ ^T-cell depletion seen in the plasma compartment was reflective of the total CD4^+ ^T-cell pool and that virus replication was driving the slow loss of cells[2]. The seemingly direct relationship of HIV-1 replication with systemic CD4^+ ^T cell loss and immunosuppression was made famous by the quote "It's the virus, stupid[3]", a humorous but pointed reference to the apparent "cause-and-effect" nature of this connection. Over the past few years, in light of new data, experts are now questioning this hypothesis.

It is now widely appreciated that both HIV-1 infection in humans, and simian immunodeficiency virus (SIV) infection in rhesus macaques (*Macaca mulatta)*, are associated with destruction of the vast majority of memory CD4^+ ^T-cells in the gastrointestinal tract in the first few weeks after infection [4-13]. Although mucosal tissues harbor a large percentage of the total CD4^+ ^T-cell population, this profound destruction is not reflected in the plasma cell pool. The depleted, mucosal CD4^+ ^T-cells are not completely replaced and the host remains deficient in memory CD4^+ ^T-cells. Some speculate that the GI tract is not unique and that a widespread mucosal immunodeficiency occurs very early after infection. In this altered state, the mucosal lymphocytes do not appropriately or adequately control invading organisms. This lack of control then contributes to a more generalized activation of the immune system, which is seen during the chronic phase of HIV-1 infection [14]. The level of immune system activation correlates with viral load and independently with the rate of CD4^+ ^T-cell depletion. Of the many activation markers, the presence of increased CD38 on CD8^+ ^T-cells correlates best with the rate of disease progression[15,16]. Many now believe chronic immune activation, not simply HIV-1 replication, leads to progressive depletion of the remaining CD4^+ ^T-cells.

Much of the new data in support of this concept comes from non-human primate models of HIV infection. More than 30 monkeys and apes are naturally infected with distinct strains of SIV [17], and most of these viruses are well tolerated by their natural hosts. As a case in point, both sooty mangabeys (*Cercocebus torquatus atys*) and African green monkeys (*Chlorocebus *spp.) are the natural hosts for SIVsmm and SIVagm, respectively. In each case, SIV replicates to high levels, but the virus does not cause circulating CD4^+ ^T-cell lymphopenia or immunodeficiency. However, experimental infection of rhesus macaques with SIVsmm, or other closely related strains of SIV, does cause a disease very similar to AIDS. Investigators have been trying to understand why SIV infection causes disease in some monkeys, but not others, as a means of unraveling the basis for immunodeficiency in humans infected with HIV-1. Reports from two recent studies now shed light on this paradox.

**Figure 1:**
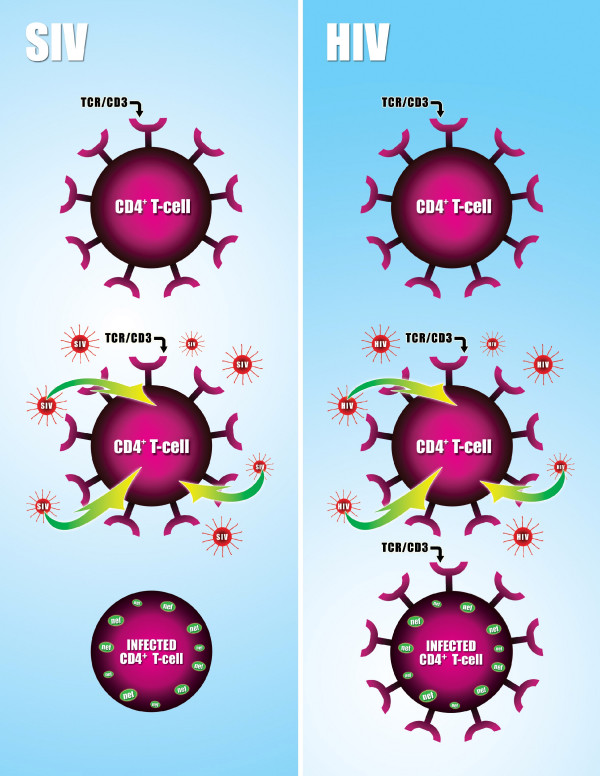
SIV (left panel) and HIV (right panel) infected CD4^+ ^T-lymphocytes. Following infection, each produces Nef, which associates with the cell membrane. However, only SIV Nef downregulates the T-cell receptor complex (TCR). HIV infected cells still express the TCR and are more prone to activation and apoptosis.

In the June issue of *Cell*, Schindler *et al. *report that *nef*, which is present in all primate lentiviruses, may protect the natural host by modulating expression of the T-cell receptor-CD3 complex (TCR-CD3)[18]. Nef is a small lentiviral protein with many attributed functions, including down regulation of CD4, CD28, and MHC-I. The authors analyzed 30 *nef *alleles from 30 different primate lentiviruses. All of the *nef *alleles down regulated CD4 and MHC-I molecules from the cell surface. Most also down regulated TCR-CD3 efficiently. However some, including those of HIV-1 and SIVcpz (a close relative of HIV-1), had no effect on TCR-CD3 cell surface expression [see Figure 1]. T-cells expressing *nef *alleles that were able to down modulate TCR-CD3 had decreased levels of activation after PHA stimulation. Further, expression of these *nef *alleles in peripheral blood mononuclear cells (PBMC) protected the cells against PHA-induced apoptosis. In contrast, those *nef *alleles that did not reduce cell surface expression of TCR-CD3 were found to increase T-cell activation and apoptosis.

The authors noted that the TCR-CD3-downmodulating *nefs *belong to viruses such as SIVsmm that do not cause disease in their natural hosts. This newly described activity of *nef *may be linked with the maintenance of an intact immune system in SIVsmm-infected sooty mangabeys. SIV plasma levels in sooty mangabeys typically exceed those of HIV-1 in humans [19-21]. Yet, sooty mangabeys do not develop immunodeficiency. By comparison, HIV-1 *nef *does not affect the TCR-CD3 complex and this may, in turn, contribute to aberrant activation of the immune system and the gradual erosion of immune function associated with AIDS.

Many questions remain regarding this hypothesis. In the chronic phase of infection, HIV-1 infects only a small minority (<1.0%) of CD4^+ ^T-cells [22], yet a much higher percentage of many different cell types possess the activated phenotype [23]. How does increased activation or apoptosis of a small percentage of CD4^+ ^T-cells (those infected with HIV-1) lead to activation of large populations of uninfected cells? Like HIV-1 *nef*, the *nef *gene of SIVcpz also does not down regulate the TCR-CD3 complex, and yet most chimpanzees infected with SIVcpz do not develop immune system activation or CD4^+ ^T-cell lymphopenia [24][25][26][27]. As discussed in the *Cell *article, SIVmac *nef *has TCR-CD3 down-regulating activity. If so, why do SIVmac-infected rhesus macaques have highly activated immune systems? Finally, what is the evolutionary advantage of HIV-1 without this activity? If the virus, which decreases TCR-CD3 expression, can replicate to high levels, how does the loss of this function of *nef *make HIV-1 more fit? Future studies are clearly needed to address these questions.

Another study of relevance to this topic was recently published in *Retrovirology*[28]. Ploquin *et al. *compared the non-pathogenic SIVagm infection of African green monkeys with the virulent infection of rhesus macaques with SIVmac. The authors measured both pro-(TNF-α and IFN-γ) and anti-(IL-10) inflammatory cytokines after *in vivo *infection. The levels of TNF-α and IFN-γ transcripts in PBMC increased significantly in rhesus macaques in the first two weeks of infection with SIVmac. In contrast, TNF-α and IFN-γ expression did not change during this time in African green monkeys infected with SIVagm. Differences were also noted in expression of IL-10, a negative regulator of inflammation, which increased in the African green monkeys at days 10–16 post-infection, but was not up-regulated in SIVmac-infected macaques. The authors found that smad4, a key intracellular, downstream signal of TGFβ-1 binding, was also up-regulated in infected African green monkeys. TGFβ-1 is an important, anti-inflammatory cytokine and these data support the hypothesis that a pro-inflammatory state is associated with pathogenic SIV infection.

Each of these new articles strongly supports the concept that immune activation, at least in part, drives CD4^+ ^T-cell depletion, whereas viremia alone is not sufficient to cause clinically significant immunodeficiency. When generalized immune system activation and viremia appear together, as in the case of HIV-infected humans and SIV-infected macaques, disease occurs. Modulation of TCR-CD3 may help prevent activation of the immune system in non-pathogenic infections, while localized CD4^+ ^T-cell depletion at the mucosal surfaces may allow antigenic stimulation and activation of the remaining cells. Pro-inflammatory cytokines are also likely to play a role: CD8^+ ^T-cells are activated and not infected with HIV/SIV, B-cells and NK cells are also activated. The level of immune activation drastically decreases with effective HIV therapy [29][30][31][32][33][34]. However, cellular activation markers do not return to normal levels even when viremia is undetectable. In most HIV-positive patients, viral suppression leads to large increases in their plasma CD4^+ ^T-cell counts. Those with little or no change in their CD4^+ ^T-cell counts generally have persistent immune system activation [35].

Using non-human primate models, researchers hope to delineate the HIV-induced immune activation pathways. Such a discovery could lead to innovative new therapies that specifically block activation of the immune system. While inhibiting HIV-1 replication during chronic infection makes sense, it is not the true goal of HIV treatment. We treat HIV to prevent or slow the development of immunodeficiency. A therapy that preserves the immune system without inhibiting virus replication would certainly be a welcome addition to currently available antiretroviral drugs that target HIV but do not adequately restore immune function.
